# Improving Care for the Frail in Nova Scotia: An Implementation Evaluation of a Frailty Portal in Primary Care Practice

**DOI:** 10.15171/ijhpm.2018.102

**Published:** 2018-11-04

**Authors:** Beverley Lawson, Tara Sampalli, Grace Warner, Fred Burge, Paige Moorhouse, Rick Gibson, Stephanie Wood, Ashley Harnish, Lisa G. Bedford, Lynn Edwards, Shannon Ryan-Carson

**Affiliations:** ^1^Department of Family Medicine, Dalhousie University, Halifax, NS, Canada.; ^2^Research and Innovation, Nova Scotia Health Authority, Primary Health Care & Chronic Disease Management, Halifax, NS, Canada.; ^3^Dalhousie University, Halifax, NS, Canada.; ^4^School of Occupational Therapy, Dalhousie University, Halifax, NS, Canada.; ^5^Health Populations Institute, Dalhousie University, Halifax, NS, Canada.; ^6^Continuing Care, Nova Scotia Health Authority, Halifax, NS, Canada.; ^7^Nova Scotia Health Authority, Halifax, NS, Canada.; ^8^Division of Geriatric Medicine, Nova Scotia Health Authority, Halifax, NS, Canada.; ^9^Palliative and Therapeutic Harmonization (PATH) Program, Halifax, NS, Canada.; ^10^Department of Family Practice, Nova Scotia Health Authority, Halifax, NS, Canada.; ^11^Primary Health Care, Nova Scotia Health Authority, Halifax, NS, Canada.; ^12^Primary Heath Care, Family Practice and Chronic Disease and Wellness, Nova Scotia Health Authority, Halifax, NS, Canada.; ^13^Chronic Disease and Wellness, Nova Scotia Health Authority, Halifax, NS, Canada.

**Keywords:** Frail Adults, Identification, Primary Care, Evaluation, Online Tool

## Abstract

**Background:** Understanding and addressing the needs of frail patients has been identified as an important strategy by the Nova Scotia Health Authority (NSHA). Primary care (PC) providers are in a key position to aid in the identification of, and response to frailty as part of routine care. Unlike singular chronic conditions such as diabetes and hypertension which garner a disease-based approach and identification as part of standard practice, frailty is only just emerging as a concept for PC. The web-based Frailty Portal was developed to aid in the identification of, assessment and care planning for frail patients in PC practice. In this study we assess the implementation feasibility and impact of the Frailty Portal by: (1) identifying factors influencing the Frailty Portal’s use in community PC practice, and (2) examination of the immediate impact of the ‘Frailty Portal’ on frail patients, their caregivers and PC providers.

**Methods:** A convergent mixed method approach was implemented among PC providers in community-based practice in the NSHA, Central Zone. Quantitative and qualitative data were collected concurrently over a 9-month period. A sample of patients who underwent assessment and/or their caregiver were approached for survey participation.

**Results:** Fourteen community PC providers (10 family physicians, 4 nurse practitioners) completed 48 patient assessments and completed or begun 41 care plans; semi-structured interviews were conducted among 9 providers. Nine patients and 5 caregivers participated in the survey. PC providers viewed frailty as an important concept but implementation challenges were met, primarily with respect to the time required for use and lack of fit with traditional practice routines. Additional barriers included tool usability and accessibility, training and care planning steps, and privacy. Impacts of the tools use with respect to confidence and knowledge showed early promise.

**Conclusion:** This feasibility study highlights the need for added health system supports, resources and financial incentives for successful implementation of the Frailty Portal in community PC practice. We suggest future implementation integrate the Frailty Portal to practice electronic medical records (EMRs) and target providers with largely geriatric practice populations and those practicing within interdisciplinary, collaborative primary healthcare (PHC) teams.

## Background

With the aging of populations in many developing countries, more attention is being focused on the provision of better care for the increasing numbers of people living longer with multiple, multisystem conditions, particularly those who are, or at risk of, becoming frail.^1,2^ Although the majority of older adults age successfully, longer life spans can result in a higher prevalence of frailty, due in part to the accumulation of multiple interacting complex health and social problems.^2-4^ Frailty often presents as a deterioration affecting mobility, cognition, function, or endurance.^5,6^ People experiencing frailty are more vulnerable to adverse events such as falls, hospitalization, disability, dependence, placement in long term care and death.^7,8^

The identification of frailty in primary care (PC) can be considered a pro-active approach.^9^ It sets the stage for better alignment between patient and family/caregiver understanding of overall health needs and their ability to make informed decisions regarding beneficial preventive strategies, medical/surgical interventions, prognosis and goals of care including end of life.^10-14^ Identifying frailty can help providers tailor interventions to help delay the progression of frailty, or at the very least, prepare patients and their families for future events and support the implementation of community-based interventions aimed at reducing risk factors.^9,12^ However, unlike singular chronic conditions such as diabetes and hypertension which garner a disease-based approach and identification as part of standard practice, frailty is only just emerging as a concept for PC with evidence-based guidelines for care being developed.^11,15,16,17^ As such routine identification and measurement of frailty has not been part of primary healthcare (PHC) regular practice and frailty often mistaken for normal aging.^4,15^

Several instruments or tools have been developed to aid the identification of frailty in PC setting.^4,18-20^ More recently the use of routine PC electronic health record data has been used in the development and validation of an electronic frailty index to identify potentially frail patients.^21,22^ However, none of these tools provide a simplified, multidimensional, approach as required in a PC setting or include an assessment to help identify the degree of frailty with associated care plan suggestions. Advances and the use of technology have enabled the development of easy, timely and relevant access and application of required tools and standards at the point of care.^9^ The use of technology has now evolved as a practical and feasible option to embed validated, standardized and relevant tools to support the application of knowledge into practice.^23^

Recognizing the challenges and opportunities to improve care for the frail, Primary Health Care and the Department of Family Practice, Nova Scotia Health Authority (NSHA) in collaboration with the Department of Medicine, and a broad group of community stakeholders began the development of what is now known as the NSHA Frailty Strategy. The Frailty Strategy outlines 6 areas of focus – understanding, engagement, care, evaluation, research and knowledge implementation, information technology and management, and governance.^24^ Together these foci aim to align new and existing frailty-focused initiatives across all organizational, community and societal sectors in the health region. The goal of the strategy is to ensure that persons of all ages experiencing frailty are supported and enabled to optimally meet the challenges of living with frailty. A primary objective of the Frailty Strategy is to identify and respond to frailty during routine care across the healthcare continuum. As most community PC providers regularly encounter patients at risk of or experiencing frailty, they are in a key position to identify and begin the planning process.

The web-based Frailty Portal was identified as a tool to meet 4 of the 6 key directions of the NSHA Frailty Strategy – understanding, engagement, care, and information technology and management.^24^ The Portal employs an electronic version of the Frailty Assessment for Care Planning Tool (FACT).^25,26^ Through a combination of patient assessment by the provider and caregiver report, the FACT, a modification of the Clinical Frailty Scale,^27^ assesses the essential domains that contribute to frailty (cognition, mobility, function and social circumstances) and provides the user with a frailty stage measure (thriving to very severely frail) for the patient. Based on the results of the FACT, the Frailty Portal then provides practical visit goals tailored to the patient’s identified frailty level, including available relevant community and online resources.

The Frailty Portal is the first web-based tool of its kind in Canada and has garnered interest from other jurisdictions across the country. In 2014, the usability of the web-based tool and the assessment component was initially piloted among a small group of PHC providers who were asked to provide their impressions of the tool. Based on their feedback modifications were made to enhance usability and the addition of visit goals and a toolkit of resources. In 2015/2016 the modified Frailty Portal was implemented among a second, cohort of community PC providers which included both family physicians and nurse practitioners. Results of that implementation are reported here.

## Objectives

In this study we assess the implementation feasibility and impact of the Frailty Portal, a web-based tool developed to aid providers in community PC practice in the identification of, and response to frailty. Our objectives were to: (1) Identify and understand factors influencing the Frailty Portal’s use in community PC practice, and (2) Assess the immediate impact of the ‘Frailty Portal’ on frail patients, their caregivers and PC providers.

## Methods

### Design

A convergent mixed methods design was used where quantitative and qualitative data were collected concurrently over a 9-month period (October 2015 to June 2016), analyzed separately, and then merged to provide a more complete understanding of the Frailty Portal’s feasibility and potential impact. Details of the study protocol have been described elsewhere.^14^

### Setting and Participants

The setting for this study was community PC practices operating within the Central Zone, NSHA in the province of Nova Scotia, Canada. Participants of the quantitative phase of this study were: (*a*) a convenience sample of PC providers (family physicians, nurse practitioners) operating under various remuneration plans and recruited through NSHA project managers, (*b*) patients of any age who had completed a frailty screening assessment with their provider and deemed cognitively able by their provider to provide informed consent and, (*c*) the patients’ family caregivers who had participated in the assessment process or were involved in subsequent discussions.

Qualitative information using semi-structured interviews were gathered from a purposive sample of PC providers (family physicians, nurse practitioners) and key PHC stakeholders (NSHA administrators, staff, decision-makers) for the main overall study. For this current article we focus on information collected from participating PC providers.

### Intervention

Components employed for the implementation of the Frailty Portal included community provider engagement, use of the web-based tool and provider supports.

*Engaging providers:* Provider participants were asked to attend a half-day face-to-face education workshop where information about the identification and care of the frail and hands-on learning of the web-based tool was received. The session was offered by research team members, composed of researchers, geriatricians, family physicians, healthcare administrators and decision-makers and PHC information technology leads. There was also support from opinion leaders, community champions and integrated community support service members (eg, home care, family and caregiver supports). One-on-one training was provided to those who could not make the session. On-going support and feedback to encourage implementation was provided by team members. Strategies included individual visits to the practice as needed to aid with the initial (or subsequent) Portal login or assessment process, telephone contact to offer additional supports and respond to questions, monthly email follow-ups and the provision of monthly individualized Frailty Portal activity reports summarizing their personal and all participants’ aggregate use of the tool. Posters to raise frailty screening awareness among patients and providers were distributed to each provider for waiting and examination room posting in each participating practice.

*Frailty Portal web-based tools:* The Portal includes tools for providers to aid the identification, screening assessment, and care/crisis planning of potentially frail patients; FACT training videos; a script to aid introducing the concept of frailty to patients/caregivers; downloadable information for patients and caregivers such as community resources, referral forms, and education materials. In brief, providers are asked to identify potentially frail patients by either reviewing their practice patient lists or through encounter-based opportunities using predetermined criterion provided within the Frailty Portal that suggest a patient may be experiencing frailty. Common characteristics associated with frailty for consideration include: an older adult with mental illness, cognitive impairment, older aged (75+ years), polypharmacy, provider gut feeling from the patients presenting issues, receiving or waiting for home care, at least 2 emergency department visits and/or at least one unplanned hospitalization, and caregiver or self-report of reduced activity, declining weight or increased exhaustion. If one or more of these characteristics for frailty exist, providers were to begin the screening process using an electronic version of the FACT^25,26^ to determine the patient’s level of frailty. Because input from the patient and their caregiver help to complete this step, providers either invited the patient and their caregiver to make an appointment to begin the process or noted in the patient chart to bring forward for discussion during their next visit. Following assessment providers were instructed to consider using suggested visit goals to aid the development of individualized care plans. An illustration of the steps involved in using the Frailty Portal, an explanation of the levels of frailty and an example of care planning goals are detailed in a previous article.^14^ Following their training, providers were asked to target the completion of at least 10 frailty assessments by May 2, 2016. Fee-for-service providers were offered an honorarium for their initial training time, however in order to reflect the ‘real life’ context of Portal use within community practice, the intervention itself was not paired with additional incentives or rewards.

### Measures

#### a) Quantitative Data

To access provider adherence to implementation activities, counts were taken to determine the proportion of those taking part in educational sessions and monthly follow-up opportunities. Through the web-tool itself data was gathered on provider utilization which included the number of patients screened and care plan activity (both partial and completed).

Prior to their education session, providers were asked to complete 2 pre-surveys. The first to collect demographic and practice information and the second a ‘Caring for the Frail’ survey to assess their pre-awareness, knowledge, confidence and satisfaction with their care for persons experiencing frailty. At study end a similar post-survey was completed. Also at study end, 2 additional assessments were administered. The ‘System Usability Scale’^28^ assessed provider experiences with the web-based tool while the ‘Post Evaluation’ survey asked questions about intervention, health system and organizational factors that could affect readiness for implementation and supports for change. The ‘Post Evaluation’ survey also captured reflections and evaluations of the process to facilitate identification of factors influencing the Frailty Portal’s use and potential scale-up. All surveys provided an opportunity for participants to add open ended comments. To maintain contact, monitor implementation, and encourage participation, monthly follow-up emails were sent to providers. The emails included a summary of their portal use and that of the collective to date, and short questions asking about their web-tool use experience, deviations in adherence, concerns and suggestions.

Eligible patients were identified by their provider and invited by mail to participate in a short telephone survey. Within each mailing were a letter addressed to the patient and a second directed toward their caregiver that included a description of their eligibility. Additionally, information about the survey and a request to provide their contact information if they would like to take part was provided. The package was addressed and mailed out by the physician and/or their administrative support team. Consenting patients/caregivers, who provided their contact information were contacted by telephone for a short survey and asked questions about their awareness of frailty and prognosis, care expectations, self-efficacy, perception of care coordination and satisfaction. An opportunity to provide comments was offered.

#### b) Qualitative Data

Semi-structured interviews were conducted among the participating providers and directed by a semi-structured interview guide to explore the implementation feasibility of the initiative and identify barriers and facilitators to integrating the portal into their practice. The qualitative analysis and findings presented here were part of a broader sample of interviews with PHC providers (family physicians, nurse practitioners) and stakeholders who were administrators, decision-makers and staff. The methods and findings from these interviews have been published separately.^29^ The findings from the sampled providers are presented in this manuscript to elucidate the quantitative results. Interviews were recorded and transcribed.

### Analysis

Quantitative data analyses were primarily descriptive. Paired *t* tests were used to assess pre-post response differences on the provider self-efficacy survey. Survey information was synthesized to aid identification of factors influencing Frailty Portal implementation and to provide a description of PC practices. Qualitative data was analyzed using qualitative description and content analysis. Qualitative descriptive analysis is used to describe rather than interpret phenomenon through an identified theoretical framework, such as phenomenology or grounded theory.^30^ In qualitative description, the researcher collects data to understand the area of study then describes this data using everyday terms as they relate to the event or area of study. Content Analysis, the process of making sense of the meanings in the data, was also used during our thematic analysis.^31^ Interviews were analyzed using NVivo software. To ensure the data was collected, analyzed and interpreted accurately, so it conveyed the experiences of participants, processes associated with trustworthiness were enacted such as member checking and reflexivity.^32^ The qualitative data from providers participating in this implementation study were triangulated to describe participant perspectives of the various aspects of implementation feasibility such as barriers and facilitators.

## Results

### Quantitative Findings

#### Provider Participants

In total 14 community PC providers (10 family physicians and 4 nurse practitioners) agreed to participate in the study. Of this group, 9 providers attended the educational workshop while the remaining 5 were provided one-on-one training sessions. Provider participants tended to be female (79%), ranged in age from 34 to 68 years (mean: 47 years [standard deviation, SD 10.2]) and practicing in urban/suburban settings (64%). Provider remuneration plans included fee-for-service (14%), service contracts (14%), salaries (29%), academic funding plans (36%) and alternative payment plans (7%). Time spent in front of patients during a typical week ranged widely from a half day to 6 days a week. Provider estimates of the proportion of their practice population that were 65 years of age and older also varied from 15% to 80% and averaged just under half (48.6%; SD 20.3). Just over half (57%) felt less than 25% of their patients (all ages) could potentially be frail. Almost all (93%) used an electronic medical record (EMR) in their practice with use ranging from 0 to 12 years.

Uptake of the Frailty Portal was slow, with only one provider completing an assessment by the end of the first 3 months. With the addition of actions to encourage Portal use, 7 providers (5/10 family physician; 2/4 NPs) completed at least one assessment by implementation end (May 2, 2016). At the end of the 8-month implementation period, an overall total of 48 patient assessments were completed and an additional 7 begun. In addition, 16 care plans had been completed and 25 in progress. Assessed patients tended to be female (73%) and ranged in age from 59 to 101 years (Mean: 81.7; SD 7.7). Varying degrees of frailty were identified (Thriving n=2, Normal Aging n = 7, Vulnerable n = 4, Mild n=14, Moderate n = 15, Severe n = 6).

Tables 1 to 3 provide the results of each provider survey. Provider perception of the ease and complexity of the Frailty Portal’s use was divided. Although responses to the System Usability Scale indicate over 70% felt the tool was easy to use and integrated well (62.5%), the majority (62.5%) were not strongly confident in using it (Table 1). Comments from the survey and those provided from monthly follow-up emails highlighted the challenge encountered in accessing and logging into the web-based tool which were primarily associated with sign in and security features that required frequent password updates. Compounding these difficulties was the lack of immediate technical support and/or a quick solution to the problem. Additional comments noted the lack of time to implement the tool within practice.

**Table 1:** System Usability Scale

**Quantitative Data: System Usability Scale**				
**Item**	**Mean (SD)**	**No. (%)**		
		**Disagree**	**Neutral**	**Agree**
a. Thought the Frailty Portal was easy to use	3.7 (0.95)	1 (14.3)	1 (14.3)	5 (71.4)
b. Found the Frailty Portal unnecessarily complex	2.3 (0.89)	4 (50.0)	4 (50.0)	0 (0.0)
c. Would like to use the Frailty Portal frequently	3.6 (1.01)	1 (11.1)	4 (44.4)	4 (44.4)
d. Would need the support of a technical person to be able to use the portal better	3 (1.73)	3 (42.9)	1 (14.3)	3 (42.9)
e. Found the various functions in the Frailty Portal were well integrated	3.6 (0.92)	1 (12.5)	2 (25.0)	5 (62.5)
f. Thought there was too much inconsistency in the Frailty Portal	2.1 (1.46)	5 (71.4)	1 (14.3)	1 (14.3)
g. Would imagine that most people would learn to use the portal very quickly	3.4 (0.92)	1 (12.5)	4 (50.0)	3 (37.5)
h. Found the Frailty Portal very cumbersome to use	2.9 (0.90)	3 (42.9)	2 (28.6)	2 (28.6)
i. Felt very confident using the Frailty Portal	3.1 (0.83)	2 (25.0)	3 (37.5)	3 (37.5)
j. Needed to learn a lot of things before I could get going with the Frailty Portal	3 (1.07)	3 (37.5)	3 (37.5)	2 (25.0)

Abbreviation: SD, standard deviation. Note: Two respondents provided their overall impression but could not respond to all items having not made full use of the tool. Scores ranged from 1 (strongly disagree) to 5 (strongly agree). However due to a limited sample size they were collapsed as Disagree (1, 2) or Agree (4, 5).

All who responded to the ‘Post Evaluation’ survey considered improving care for their frail patients a high priority, an important concept for their practice and a better way to identify frail patients in their practice in comparison to other clinical evaluations currently being used (Table 2). Most agreed that it aligned with an identified clinic need (87.5%) and use of the tool could help improve communications with their frail patients (88.9%). However, all also indicated issues associated with time, the tool did not fit easily into appointment time (100.0%), took too much time to use (66.7%) and did not fit with the way they conducted patient visits (66.7%). Although all felt the implementation was well organized, most felt the implementation could benefit from the provision of a practice facilitator (83.3%) and all agreed the tool would be better used if it was linked with their practice EMR. Several comments were provided to support these responses. Among providers who are members of a clinical team, only half felt there was support from others in their clinic/practice to continue using the Frailty Portal following the required study period. Less than half (44.4%) felt their clinic had enough resources (eg, people, time) to continue (Table 2).

**Table 2:** Provider Perceptions of the Usefulness of the Frailty Portal tool

**Quantitative data: Evaluating the Frailty Portal**			
**Items**	**Mean (SD)**	**No. (%)**	
		**Disagree**	**Agree**
**The Frailty Portal …**			
a. … is a better way to identify frail patients than other clinical evaluations I could use	2.4 (0.53)	0 (0.0)	9 (100.0)
b. ... improves my ability to care for frail patients	2 (0.87)	1 (11.1)	8 (88.9)
c. … aligns with a need identified by me or my clinic	1.9 (0.35)	1 (12.5)	7 (87.5)
d. … fits easily into appointment times	0.3 (0.50)	9 (100.0)	0 (0.0)
e. … takes too much time to use	1.7 (0.87)	3 (33.3)	6 (66.7)
f. … complements other services provided in my practice	1.9 (0.78)	3 (33.3)	6 (66.7)
g. … could reduce unnecessary hospital use by assessing frail patients earlier	2 (0.53)	1 (12.5)	7 (87.5)
h. … fits well with the way I conduct patient visits	1.2 (0.97)	6 (66.7)	3 (33.3)
i. … can improve communications with my frail patients	2.3 (0.71)	1 (11.1)	8 (88.9)
j. … implementation was well organized	2 (0.0)	0 (0.0)	8 (100.0)
k. … would be better used if it was linked in some way to our practice EMR	2.8 (0.44)	0 (0.0)	9 (100.0)
**Provider Opinions**			
l. Improving care for my frail patients is a high priority for me	2.6 (0.52)	0 (0.0)	10 (100.0)
m. I support the use of the Frailty Portal in PC practice	2.2 (0.79)	2 (20.0)	8 (80.0)
n. I believe frailty is an important concept for my practice	2.7 (0.48)	0 (0.0)	10 (100.0)
**Asked of Providers Who Are Members of a Clinical Team**			
o. The patient information from the Frailty Portal is discussed with appropriate team members to improve care	1.6 (1.00)	3 (42.9)	4 (57.1)
p. There is support from others in the practice/clinic to continue using the Frailty Portal after this pilot	1.3 (1.16)	4 (50.0)	4 (50.0)
q. Our clinic has enough resources (eg, people, time) to continue to use the Frailty Portal after the pilot	1.3 (1.00)	5 (55.6)	4 (44.4)
r. Our practice/clinic is comfortable trying new programs	2 (1.12)	3 (33.3)	5 (66.7)
s. The team debriefs on ways to improve services for frail patients	0.8 (0.71)	7 (87.5)	1 (12.5)
t. Providing a practice facilitator to help integrate the Frailty Portal into my practice would have been beneficial	2.7 (0.82)	1 (16.7)	5 (83.3)

Abbreviations: SD, standard deviation; EMR, electronic medical record; PC, primary care. Note: Scores ranged from 1 (strongly disagree) to 4 (strongly agree). However due to a limited sample size they were collapsed as Disagree (1,2) or Agree (3, 4).

The provider self-efficacy ‘Caring for the Frail’ survey was implemented pre and post Frailty Portal implementation. Nine providers of the initial 14 completed both the pre and post surveys. At study end, all mean confidence scores had increased to some degree (Table 3). Although the number of providers with matched scores was small, a statistically significant increase was evidenced with respect to having greater confidence in their ability to assess the degree of frailty among patients (*P* = .04) and discussing with patients that their combined health issues have put them at risk for being frail (*P* = .01). Reasons for lower levels of confidence offered by providers within the survey included the lack of special education in Canada addressing frailty, lack of experience with frail patients, limited communication skills about the subject, not wanting to discomfort patients with a label and a lack of care planning supports and resources.

**Table 3:** Provider Confidence in Caring for the Frail Pre/Post Results

**Quantitative Data: Caring for the Frail Survey**			
**Item**	**Confidence Score** **Mean (SD)**		**Mean Paired Difference (n = 9):** ***P*** ** Value**
	**Pre (n = 14)**	**Post (n = 9)**	
I am confident I have a:			
a. Comprehensive understanding of frailty	2.21 (0.58)	2.44 (0.24)	.51
I am confident I have the ability to:			
b. Identify frail patients in my practice	2.42 (0.85)	2.67 (0.87)	.64
c. Assess the degree of frailty among my patients	1.79 (0.80)	2.89 (0.78)	.04
d. Explain and provide information on frailty	1.85 (0.77)	2.44 (1.13)	.24
e. Discuss care options with my frail patients	1.93 (0.62)	2.44 (1.13)	.35
f. Discuss care options with my frail patients’ family/friend/caregivers	2.0 (0.68)	2.33 (1.22)	.52
g. Engage frail patients and their family/friend/caregivers in the decision-making process	2.14 (0.77)	2.56 (1.01)	.21
h. Develop service care plans reflective of my frail patients and their caregiver needs and concerns	1.86 (0.66)	2.22 (1.09)	.66
i. Identify relevant community resources for my frail patients	1.6 (0.63)	2.11 (1.17)	.35
j. Refer frail patients to appropriate community resources	1.71 (0.61)	2.44 (1.13)	.17
k. Coordinate access to needed services	1.57 (0.65)	2.11 (1.05)	.27
Degree of confidence felt:			
j. In using the word frail	2.21 (0.80)	2.56 (0.88)	.31
m. Discussing with patients that their combined health issues have put them at risk for being frail	2.0 (0.78)	2.89 (0.93)	.01
n. Discussing the degree of frailty and next steps with my frail patient and their caregivers	1.92 (0.73)	2.44 (1.13)	.22

Abbreviation: SD, standard deviation. Note: Confidence is rated from 1 (not very confident) to 4 (very confident).

Providers remained relatively non-confident in using the term ‘frail’ when speaking with patients and their caregivers. Reasons provided in the comments for not using the term included concern for patient comfort, and patient knowledge of, and resistance to the term. Providers noted talking around the issue and not giving it a name or commented they were ‘not as strong as they used to be.’

#### Patients/Caregiver Participants

The very short time frame of the study combined with the delay in the implementation process by providers and need for patient selection and communication to originate from the providers office resulted in patient/caregiver recruitment challenges and gaps in knowledge about the patient and/or their caregiver. In total 25 eligible patients and their associated caregiver (if applicable) were mailed an invitation to participate in the telephone survey. Of a possible maximum of 50 respondents (25 patients; 25 caregivers), 9 patients and 5 caregivers consented to take part (response rate: 28%). Gaps in knowledge about patients and caregiver participants included individual demographics, identified frailty level, the provider who performed the assessment, whether patients had a caregiver and, for those who responded, the relationship between a patient and caregiver.

Following their assessment, all patient respondents (100%) indicated they knew more about how to care for themselves and were happy that their doctor or nurse talked with them about frailty and ways to self-manage their care. Most (88.9%) felt they now had a better understanding about how frailty can progress and how to find help and required information about living with frailty. There still appeared to be some doubt among patients in whether they knew more about their current health status; 44.4% indicated not gaining any additional knowledge following provider discussions. Unsolicited comments indicated some patients did not see themselves as frail and others noted their provider would ‘talk around’ the subject of frailty by discussing individual conditions rather than being direct.

Caregivers all indicated that they had a better understanding of the importance of caring for themselves as they continued to care for their family member. Most (80%) felt they had a better understanding of what to expect as the patient in their care continued living with frailty and were involved in the decision-making process as much as desired. Just over half of caregivers (60%) felt they had a plan in place to help deal with a health emergency. Although 75% of caregivers indicated being better prepared to care for their family member, more than half (60%) felt they did not know more about frailty than before the assessment. Within the comments caregivers indicated confusion with the form they were provided to record information to aid the assessment process and the need for printed information to be available in the waiting room.

### Qualitative Findings

Semi-structured interviews, lasting 40 to 90 minutes, were conducted among 6 family physicians and 3 nurse practitioners, some of whom applied the Frailty Portal in their practice and others who did not or had limited use. For the full qualitative analysis PC provider data was combined with information provided by 8 PHC stakeholders where qualitative descriptive and content analysis identified 3 themes (1) PHC Practice Context, (2) Intervention attributes affecting implementation, and (3) Targeting providers with frail patients. These findings have been presented in a separate publication.^29^ For this manuscript we focus on content areas within these themes identified as influencing the implementation feasibility of the Frailty Portal within PC practice by providers of this implementation study. Overall the Frailty Portal was viewed positively, despite the multiple challenges to implementing it. Difficulties in accessing the web-based portal and the need for the tool to be integrated with the practice EMR was commonly voiced. Providers perceived high opportunity costs to using the Frailty Portal due to changes they needed to make to their practice routines and the time required to use the tool from assessment to care planning. However, those who had older patients, took the time to learn how to use the Frailty Portal, and created processes for sharing tasks with other PC personnel become proficient at using the Frailty Portal. Quotes from the provider interviews have been extracted from these content areas to triangulate the qualitative data with the quantitative data in alignment with a convergent mixed methods analysis (Table 4).

**Table 4:** Major Integrated Findings

**Key findings**	**Quantitative Support**		**Qualitative Support Interviews**	**Implications**
	**Survey Responses**	**Survey Comments**		
Concept of frailty	All (100%) believe frailty to be an importance concept for their practice and improving care for their frailty patients a high priority. (Table 2 Items n, l)	None	*“It makes perfect sense and it fits right into with what we’re doing … disease prevention, health promotion, chronic disease management …”* [HP6-I].	Providers value the concept of frailty and believe it is important to address with their patients. If providers can fit the tool into their routine practice there should be good uptake.
Frailty Portal usability and accessibility	71.4% felt the tool was easy to use (Table 1 Item a)	*“… lots of difficulty logging in”* [HP7]. *“I thought the portal was well put together and easy to use”* [HP10].	*“Oh, it’s very easy [to use the portal] … training day it was perfectly understandable …. But I never got the opportunity to do that with a client sitting in front of me because of the difficulty that I had logging on”* [HP1].	Providers need easy access to the Frailty Portal. Removing the need to log in appears to be one of the major obstacles to using the tool. Changing login procedures are being investigated.
	100% felt it should be linked to the practice EMR (Table 2, Item k)	*“It needs to be tied to the EMR”* [HP5-S].	*“If it was embedded somehow or operationally more integrated into the EMR that would be great”* [HP2].	Based on feedback, the Frailty Portal should be integrated into the EMR. Integrating the tool into the EMR is being investigated.
	44.4% responded they would like to use the Frailty Portal frequently (Table 1, item c)	None	*“… you have to have a certain number of older [patients] … it’s obviously something for the older population. … the more patients you have, the more likely you’ve got some older [and to use it]”* [HP4].	Providers with a more elderly practice population would have greater opportunity for use and should be targeted for future implementation.
**Time and practice organization**	100% felt the Frailty Portal did not fit easily into appointment times (Table 2, item d)	*“My problem with getting started using the frailty portal is/was time. I do not have extra time in my schedule to accommodate”* [HP10].	*“It’s not a 15-20 minute appointment. And that’s what we have. You know, so it’s hard to incorporate it into my day-to-day routine”* [HP1].	Providers need adequate time to use the tool. It is important to implement supports for adequate appointment times.
	Less than half (44.4%) felt their clinic had enough resources to continue using the Frailty Portal after the pilot (Table 2, item q)		*“I think many of us … aspire to be innovative but practically don’t feel we have a lot of resources to be that way…”* [HP3]. *“Certainly if you were in a team, you could farm out the activity”* [HP8].	Additional practice resources aiding the process would be helpful. Interdisciplinary, collaborative practices may be better able to support Frailty Portal use.
	66.7% felt it took too much time to use (Table 2, item e)	*“Time factor limited my use”* [HP12].	*“… it would be a great thing … but what is the opportunity cost to [physicians] of doing that [using the Portal] versus something else?”* [HP6].	Same as above.
	66.7% felt it did not fit well with the way they conduct patient visits (Table 2, item h)	*“It does take a bit of time and I found it better suited to being a focus of a visit rather than an addition to an office visit”* [HP10].	*“The challenge is in encounter/structure with a patient in which you can apply the tool...”* [HP2].	Pre-identification of potentially frail patients and scheduling for frailty specific appointments is suggested.
		*“For widespread implementation a higher billing code would encourage use as it takes a long time to complete”* [HP13].	*“… fee-for-service, there has to be some mechanism to be paid appropriately for the amount of time it takes…”* [HP4].	An appropriate fee schedule for fee-for-service reimbursement is recommended.
Training and steps following assessment	Most (83.3%) felt a practice facilitator to help integrate the Frailty Portal into my practice would have been beneficial (Table 2, item t)	*“…need practice change facilitator & support”* [HP-5].	*“… if the practice support person had already been in place … it would have probably allowed the Frailty Portal to have been integrated more smoothly”* [HP4-I].	Future implementation could potential benefit from practice facilitation.
	Provider confidence in discussing the degree of frailty and next steps with frail patient and their caregivers significantly improved but remained relatively low to moderate (Table 3, item n)	*“More skills related to care planning would be beneficial … how to discuss these topics with families …”* [HP9]. *“I think it identifies frailty well but I am not sure it really helps next steps”* [HP11].	**“** *Collecting the collateral information is usually a barrier and then working through all of the suggestions (care options) is huge”* [HP11].	Providers require greater confidence with the concept of frailty. Several new online educational modules and videos are being developed to support providers in their use of the Frailty Portal.
Use of the term ‘frail’	Providers remained relatively non-confident in using the term ‘frail’ (Table 3, item j)	*“I feel that patients may not be comfortable with this label”* [HP10]. *“I would talk around it without giving it a name – discuss number of medical issues, mobility concerns etc without labelling”* [HP3].	*“…frailty was sort of unspoken or unrecognized. So that you were looking at a frail patient but you didn’t label them as frail. And not having labeled them as frail, you kind of forgot to manage the frailty”* [HP8].	Same as above.

### Integrated Findings

Table 4 summarizes the key integrated findings and implications for improvements. Although quantitative and qualitative finding support the concept of frailty and provision of better care to frail patients as important to providers, the integrated results highlight factors, both barriers and facilitators, which influenced their ability to implement the ‘Frailty Portal’ in community PC practice.

### Factors Influencing the Frailty Portal’s Use in Community Primary Care practice

#### Barriers

The quantitative results indicated possible barriers to the uptake of the tool that were further elucidated by the qualitative survey comments and interviews. The major integrated findings were:

#### Frailty Tool Usability and Accessibility

Provider perception of the ease and complexity of Portal use was divided. For many the initial step of accessing or ‘logging’ into the web-based Portal was a big enough issue to discourage use. These issues were primarily associated with sign in and security features that required frequent password updates which were often compounded by a lack of immediate technical support and/or a quick solution to the problem. Currently the Frailty Portal is not integrated with a practice’s EMR and required separate logins and computer screens. This was problematic when the provider wanted to refer to the patient’s record while using the Frailty Portal. As well, the results of the Frailty Portal did not automatically become part of the patient’s practice record. Participants expressed a need to integrate the Portal into the EMR. Providers who used the tool more frequently were more confident in its use. Practices with a more aged patient population tended to have greater opportunity to use the tool.

#### Time and Practice Organization

Lack of time was expressed for all steps in using the Frailty Portal: the identification of potentially frail patients for assessment, the assessment process itself and follow through with care planning components. Its’ use did not fit into allocated appointment times or how encounters were routinely structured and some expressed pressure in their not being able to see more patients in the same time slot. The lack of, or confusion of the existence of a billing code or reimbursement strategy to compensate for the extra time required to use the tool was viewed as a barrier for some. Support from others in their clinic/practice to continue use of the Frailty Portal following the required study period and the resources to do so was believed to be lacking. However, it was noted that interdisciplinary collaborative care teams, where care is often shared, are better positioned to implement the Frailty Portal in practice.

#### Training and Steps Following Assessment

Providers noted a lack in practice resources or supports for clinical change, such as a practice facilitator or visible system champions to provide encouragement for their efforts, advice in using the tool or suggestions on how best to structure practice visits for frailty focused encounters. Most found their initial training session effective and the Frailty Portal valuable in aiding the identification of potentially frail patients and their ability to assess patient level of frailty but desired more follow-up and support, particularly with respect to initiating discussions of frailty with patients and their caregivers, the development and implementation of care plans and details about available community resources. Some expressed difficulties in obtaining information from the caregiver or family (the ‘collateral’) for the assessment process and not being familiar with community resources that were listed, a resource that was found to be underutilized by participants.

#### Privacy Concerns

Concern was expressed by providers about approaching their patients and a family member for an assessment even though it would be beneficial for them. Others questioned the ethics of having practice staff help pre-identify potential frail patients for provider review and then calling them to pre-arrange an appointment for assessment.

#### Facilitators

Some providers found it very helpful to first pre-identify potentially frail patients and pre-schedule a visit for assessment purposes. This step tended to work best where practice/clinical resources were available. However, as noted above, others raised concern about privacy. Overall an interdisciplinary practice environment where family physicians, family practice nurses and/or nurse practitioners worked collaboratively was better able to support the use of the Frailty Portal as were providers with a larger, older practice population. Suggestions provided to facilitate use included aiding a prompt to the EMR to screen for frailty, real time technical support and a platform for delivery and reimbursement.

#### Impact of the Frailty Portal

Qualitative interviews among providers were focused primarily on the feasibility of using the Frailty Portal and not the impact of its use. With the exception of using the term ‘frail’ with their patients, there was limited information to merge with survey results (Table 4). Mixed results support the usefulness of the tool with respect to its’ value in the identification and assessment of frail patients and the need for additional care plan training. Survey comments and interview data provided insight into why providers remained relatively non-confident in using the term ‘frail’ with patients and their caregivers. Most reported talking in general terms around frailty and focused on other medical conditions to avoid labelling the patient. The frail label was viewed negatively in general and felt could result in patient discomfort. Patients and/or their caregivers did not participate in qualitative interviews.

## Discussion

Identified barriers, facilitators and early impacts are intermixed and essentially tell the same story. Frailty is an important concept in PC practice and providers have the desire to improve care for their frail patients. However, even in the context of this implementation study, they faced challenges in making full use of the Frailty Portal. For some it was an issue of easy access, for others following through with the various care planning components; but for most, use of the web-based Portal did not fit into their regular practice routine and took too much time which resulted in missed ‘opportunity’ to see other patients. These issues combined with a lack of resources or supports to aid the process or a change in reimbursement structure, limited provider uptake.

The most important factor potentially affecting implementation feasibility within participating PC practice settings is how PC practice in Nova Scotia is structured and the pressures providers felt to prioritize their time to see as many patients as possible. Increased time to care for complex and frail patients is counterintuitive to most PC practice settings where high volumes of patients are typically seen within shorter appointment time allotments. Although the number of interdisciplinary collaborative practices in Nova Scotia is increasing, most community practices are essentially fee-for-service private businesses where the provider bills the province for their medical services. In general, participating nurse practitioners associated with collaborative team practices felt the tool more compatible with their work as it aligned with longer appointment times and management of chronic conditions (frailty being one of them).

The Portal existed outside of the practice EMR and required logging into a system that proved difficult for some – all adding time to the patient encounter. For successful implementation the process must be quick, simple to perform and with no barriers to tool access. Experiencing technical issues during an encounter with a potentially frail patient will only discourage future use. Those who learned to use the Portal and integrate it into their practice routines became proficient and tended to use the tool more regularly. We highly recommend future implementation of the Portal in PHC be integrated with practice EMRs. Doing so would be a positive step toward simplifying the access issue and potentially lead to a reduction in the time associated with its use.

The need for added supports, resources and financial incentives for successful implementation of a complex intervention in community PC practice are highlighted in this feasibility study. The web-based tool was felt to be relatively easy to use and navigate but the time required for use proved to be incompatible with most practice routines. The need for practice facilitation to help integrate the intervention into practice and ‘make it fit’ within the structure of their practice and patient visit routines was strongly voiced. Practice facilitation within PC is known to have a moderate effect on the uptake of evidence-based guidelines and prevention activities.^33^ Although the need for practice facilitation was identified prior to implementation, unforeseen circumstances prevented our doing so. This support would be beneficial in future PC implementation strategies.

Providers indicated that fair financial compensation for the extra time required to become familiar with and implement the various components of the frailty Portal was needed. However the effectiveness of financial strategies to improve change in PC has been reported to be mixed and context specific.^34^ How best to compensate for the extra time required should be investigated.

Implementation of interventions within the PC setting are recognized as being very complex and tend to be require change at multiple inter-dependent levels.^35^ As previously noted, most community PC practices in Nova Scotia are fee-for-service private businesses. NSHA can engage providers and support their health service delivery as well as their participation in health authority driven initiatives, such as the Frailty Portal, but are not able to mandate programs or easily provide internal supporting resources. As such without a strong organizational structure, implementation of any intervention broadly across community practice is very challenging.

A general lack of cohesion between PC providers can lower the effectiveness of peer pressure and organizational culture which have been demonstrated as effective models to stimulate change in other practice contexts, such as within acute care settings. For example, peer pressure has been associated with improved patient flow within emergency departments, improved use of advanced imaging tests for women with breast cancer and better hand hygiene.^36,37^ In contrast, audit and feedback, a form of peer pressure used in this study by providing monthly follow-up emails and a summary of their Portal use as well as that of all participating providers, had little effect on Portal utilization. Instead our result lends support to that reported in a 2015 systematic review where peer pressure strategies were found to be relatively ineffective in achieving change in PC.^34^ Some have suggested that such strategies may have greater influence within practices composed of interacting interdisciplinary teams with support personnel.^38^

Presently there is a need for both patients and providers to value frailty screening as a normal part of healthcare for aging adults. Because there will always be trade-offs for busy providers when choosing how best to use their limited time, the justification for spending added time to use the Frailty Portal needs to be clearer. An additional training component stressing the benefits of identifying frail patients in their practice and how best to proceed with the suggested care planning steps would be best combined with change management supports and reimbursement for the activity. It is acknowledged that providers with practice populations with a larger proportion of potentially frail patients might view the opportunity cost to using the Frailty Portal as more beneficial. As such we suggest targeting these practices for future implementation.

Due to the many challenges associated with provider uptake of the Frailty Portal and the short implementation period, the impact of Portal use was only just emerging. Early results suggest the introduction of the Frailty Portal to community PC practice resulted in increased provider confidence in initiating conversations with patients and their caregivers about frailty and in their ability to articulate how health issues can combine to put them at risk for being frail. Providers also felt better able to assess the degree of frailty among their patients and discuss care plan options. However, the discomfort of using the term ‘frail’ with their patients and/or their caregiver remained with many preferring to talk about the patients’ multiple health issues or the patient ‘not being as strong as they used to be.’ Recent studies confirm the negative connotation of being labelled as frail among older adults.^38-41^ Although Schoenborn et al found that frail participants in their study had a greater desire to discuss their frailty with providers than non-frail participants, all would prefer the term ‘frail’ not be used.^40^ The need to develop a more acceptable term or way to discuss frailty in practice was suggested by the authors.^40^

Although very limited in number, most participating patients indicated gaining a better understanding about the progression of frailty and caregivers a better understanding of what to expect as they care for their frail family member. These results are very encouraging given the limited time frame for implementation and evaluation. However how best to approach patients and/or their caregivers given the need for confidentiality requires further investigation.

## Limitations

Several limitations were associated with this implementation feasibility study. Although providers were initially enthusiastic following the education sessions, they were very slow to try out the Frailty Portal in their practices. This time lag resulted in the late identification of technical problems accessing the web-based tool from their practice site which often necessitated finding time in their busy schedules for on-site help by a member of the team. This issue could potentially have been avoided or reduced if a practice facilitator responsible for aiding implementation of the tool into practice was available. In addition, the time lag potentially contributed to fewer patient assessments and care plans being completed by those who began the process late and others who gave up feeling it was too late to start. The slow uptake also compounded what was already a very short timeline, particularly with respect to the assessing the immediate impact of the intervention among patients and/or their caregivers who, due to privacy concerns, were identified as eligible and invited to take part in a short survey interview by the provider and/or their practice. It is possible that, for some providers, not offering a financial incentive to compensate for the additional time required to identify, assess and begin care planning for frail patients affected their uptake. As identified by providers, steps following the assessment of potentially frail patients such as the discussion of care planning goals and community supports were newer additions to the Frailty Portal and potentially not as well developed or supported as required.

## Conclusion

The implementation of the Frailty Portal within community PC practice is representative of a complex, transformative, health system intervention. Not only are the needs of the patient and their caregiver multifaceted and complex, but the context of PC practice itself as well. Our assessment of the feasibility of implementing the Frailty Portal in community PC practice has aided the identification of challenges contributing to the successful uptake of the tool which ranged from initial access difficulties to the lack of time available and supports to undertake each step within traditional PC practice. It is suggested that future implementation target practices with an older practice population and those practicing within interdisciplinary collaborative team environment. Practice supports to help design and facilitate change, resources to aid practice identification of potentially frail patients and help follow through with care plan goals, and re-imbursement strategies are also recommended. In addition, ease of access to the web-based Portal is imperative as is the consideration of integration with PC practice EMRs and additional training opportunities. Implementing new innovations takes time, which must be respected. As such barriers identified by this evaluation are not unexpected and several may have been resolved if provided a longer follow up period to track implementation. Future research is encouraged to help identify how best to facilitate change within PC practice and models that can support the complexity of care for frail patients and their caregivers in the community.

This evaluation highlights the need for added supports, resources and incentives to be in place for future PC innovations to enable providers to execute their role in addressing the growing need of whole person or holistic models of care for frail Canadians at the community level.

## Ethical issues

Ethical approval was received from the NSHA Research Ethics Board.

## Competing interests

TS, SW, AH, LGB, LE, RG, SRC, and PM all work for the Nova Scotia Health Authority (NSHA) and were either involved with the creation of the Frailty Portal and/or work for the Department that was.

## Funding

This project was funded by the Canadian Frailty Network (TVN FRA2015-B-17).

## Authors’ contributions

Conception and design: BL, TS, GW. Drafting of the manuscript: BL, GW, SW, TS. Critical revision of the manuscript for important intellectual content: FB, RG, PM, SW, LGB, AH, LE, SRC. Obtaining funding: BL, TS, PM, LGB, FB, RG, GW. Administrative, technical, or material support: SW.

## Authors’ affiliations

^1^Department of Family Medicine, Dalhousie University, Halifax, NS, Canada. ^2^Research and Innovation, Nova Scotia Health Authority, Primary Health Care & Chronic Disease Management, Halifax, NS, Canada. ^3^Dalhousie University, Halifax, NS, Canada. ^4^School of Occupational Therapy, Dalhousie University, Halifax, NS, Canada. ^5^Health Populations Institute, Dalhousie University, Halifax, NS, Canada. ^6^Continuing Care, Nova Scotia Health Authority, Halifax, NS, Canada. ^7^Nova Scotia Health Authority, Halifax, NS, Canada. ^8^Division of Geriatric Medicine, Nova Scotia Health Authority, Halifax, NS, Canada. ^9^Palliative and Therapeutic Harmonization (PATH) Program, Halifax, NS, Canada. ^10^Department of Family Practice, Nova Scotia Health Authority, Halifax, NS, Canada. ^11^Primary Health Care, Nova Scotia Health Authority, Halifax, NS, Canada. ^12^Primary Heath Care, Family Practice and Chronic Disease and Wellness, Nova Scotia Health Authority, Halifax, NS, Canada. ^13^Chronic Disease and Wellness, Nova Scotia Health Authority, Halifax, NS, Canada.

## Key messages

**Implications for policy makers**

- The implementation of new strategies within community primary care (PC) practice is very complex due to the context of PC practice itself and the multifaceted complexity of the needs of patients and their caregivers.
- Frailty is an important concept in PC practice and providers desire to improve care for their frail patients.
- How PC practice is traditional structured can be a barrier to uptake of innovations due to the additional time required and the need to see as many patients as possible.
- Uptake is better aligned with practices composed of a more elderly patient population and interdisciplinary collaborative team practices.
- Successful implementation strategies would benefit from the addition of practice resources and supports to design and facilitate change, continuing education and reimbursement strategies for the additional time required.

**Implications for public**

Helping patients live optimally across the lifespan is essential to primary care (PC) providers. We explored how an online Frailty Portal screening tool could help PC providers identify, determine the level of frailty of and begin care plans for frail patients of all ages in order to help keep them as healthy as possible. After using the tool, providers reported more confidence in their understanding of frailty, ability to assess the degree of frailty among their patients and discussing with patients their combined health issues that put them at risk for frailty. While PC providers wanted to use the tool, results indicate that using it in their practice setting required longer appointment times than traditionally offered and that they would require help in the practice to follow through with the care planning steps. Overall, the Frailty Portal offers providers with a systematic way to think of frailty, assess patients and include patients and families into the conversation. With improved PC supports and more collaborative working environments, tools like this can be implemented to improve care.
